# Cross-kingdom small RNA communication between plants and fungal phytopathogens-recent updates and prospects for future agriculture

**DOI:** 10.1080/15476286.2023.2195731

**Published:** 2023-03-29

**Authors:** Bijayalaxmi Mahanty, Rukmini Mishra, Raj Kumar Joshi

**Affiliations:** a Department of Biotechnology, Rama Devi Women’s University, Bhubaneswar, Odisha, India; b School of Applied Sciences, Centurion University of Technology and Management, Bhubaneswar, Odisha, India

**Keywords:** Fungal sRNA, plant mirnas, RNA interference, plant immunity, extracellular vesicles

## Abstract

Small RNAs (sRNAs) are short non-coding regulatory RNA sequences that silence the complementary expressive transcripts through an endogenous RNA mediated interference mechanism (RNAi). These sRNAs typically move through plasmodesmata and phloem in plants to support disease resistance, and also through septal pores and vesicles in fungi to act as effector of pathogenicity. Notably, recent reports have shown the occurrence of a bidirectional trafficking of these sRNAs between the host plants and the attacking fungal phytopathogen which have significant implication in the nature of the infection. While the trans-species sRNAs from the pathogen can silence the host mRNAs and inhibit the host immunity genes, the sRNA modules from the host plants can silence the mRNA in the pathogen by impeding the expression of the pathogenicity-related genes. In the present review, we discuss the current state of sRNA trafficking between the plant and the pathogen with special emphasis on the mechanism of cross-kingdom communication which could contribute to the development of pathogen and pest control in future agriculture.

## Introduction

Eukaryotic small RNAs (sRNAs) are 20–30 nucleotides short functional regulatory RNA molecules that are implicated in growth, development, metabolism, genome integrity, and plant–pathogen interaction [1]. sRNAs are widely spread in eukaryotes and regulate the gene by transcriptional gene silencing or post-transcriptional gene silencing, through the creation of mRNA break, target inhibition, and/or DNA methylation [2,3]. Among other, the major functions of plant small RNAs are to inhibit infections of pathogens and pests by inducing RNA interference (RNAi). RNA interference is a conserved, negative regulatory mechanism in eukaryotes and plays a pivotal role in growth, development, and host–pathogen interaction. The sRNAs loaded with the endonuclease or DICER-like protein bind to AGO and degrade the passenger strand while the guide RNA binds to the RNA-induced silencing complex (RISC) to target and silence the complementary mRNA sequence to control the expression of genes at the transcription and/or post-transcription level [4]. Previously, it was believed that miRNA is non-existent in fungi. However, accumulated evidences have shown that RNAi mechanisms have a conserved, defensive, and regulatory role in maintaining genome integrity in fungi. The advent of high throughput sequencing in various ascomycetes and basidiomycetes, has led to the identification of miRNA-like RNAs (miLRNAs) similar to those found in plants and animals [5–7]. After a successful fungal infection, the defence machinery of the plants is hijacked by the pathogen using these sRNAs [8].

Plant-derived sRNAs can be transported to fungus as naked sRNAs, or bound to RNA-binding proteins (RBP), or enclosed inside the extracellular vesicle [9]. Plant sRNAs often target the silencing of fungal genes encoding for virulence, toxin production or pathogenesis. Contrary to this, fungi can also transport sRNAs to plants for silencing the defence mechanism. These sRNAs can move through vesicles or by binding with RNA binding protein or in naked form to induce pathogenesis [9]. While the extracellular vesicle (EV)-mediated sRNA transport has been reported in *Botrytis cineria* [10], other studies have demonstrated that the majority of exRNAs are not bound to EVs [11]. The analysis of the apoplastic wash fluid (AWF) purified from Arabidopsis leaves have shown that the extra-cellular naked sRNAs located outside the vesicles contribute to host-induced gene silencing [11]. Based on this, the pathogen-derived sRNAs could be divided into two types: − 1) endogenous sRNAs that help in activating the virulence gene during pathogenesis, and 2) sRNAs that transport into the host cell and silence the plant immunity genes thereby acting like a sRNA effector [12]. This deployment of sRNA between plants and fungi through plasma membrane for silencing the virulence gene(s) in the pathogen or the defence responsive gene(s) in plants by RNAi approach is known as cross-kingdom communication. The movement of cell non-autonomous sRNAs between two plant cells is a short-range communication that occurs through the desmotubule of plasmodesmata [12]. However, the long-distance movement of sRNAs from top of the shoot to the bottom of the root is mediated by a phloem sieve tube [13]. Mobile miRNAs have shown clear characteristics of a signalling molecule for plant development and stress response as evidenced from the cellular transportation of miR399 during phosphate starvation in plants [14]. Alternatively, some plants can release packaged sRNAs outside the cells with the EV to be absorbed by a fungal pathogen [15]. Such EV mediated cross-kingdom targeting mechanism has been detected in many plants including tomato [16], and sunflower [17]. Interestingly, these EV-coated sRNAs are transported into pathogenic fungi to inhibit the pathogenic responsive genes in *Gossypium hirsutum* and *Cuscuta campestris* [18,19]. On the other hand, a wide range of pathogenic fungi such as *Botrytis cinerea* [20,21], *Verticillium dahlia* [22], *Fusarium graminearum* [23], *Hyaloperonospora arabidopsidis* [24], *Blumeria graminis f. sp. tritici* [25] and *Fusarium oxysporum f sp. lycopersici* [26] transport sRNAs to plants and silence the resistance mechanism. Therefore, it is essential to understand the mechanism of sRNA movement across the plant and pathogen to exploit them for agricultural benefits.

In the recent time, several RNAi-based disease prevention mechanisms have been recommended for crop protection. This includes host-induced gene silencing (HIGS) [27], spray-induced gene silencing (SIGS) and nanocarrier-based spray-induced gene silencing (nSIGS), which are considered eco-friendly, stable, efficient, and sustainable strategies for protection against plant infection. In HIGS, dsRNAs and siRNAs are generated from the transgenic plant, which act upon the eukaryotic pathogen or pest. Likewise, in SIGS and nSIGS, the silencing effect is realized through exogenous spray of dsRNAs and siRNAs on the topical part of leaves infected with the pathogen [28]. Also, the CRISPR/Cas9 system has emerged as one of the most novel approach for silencing of host genes and/or the pathogenic genes to regulate the host–pathogen interaction [29,30]. In this review, we have made a comprehensive discussion about the mechanisms of sRNA communication between plants and phytopathogenic fungi with special emphasis on EV mediated transport of sRNAs. We have also discussed about the utilization of various RNAi based approaches and their utilization in the development of crop protection strategies.

## Cross kingdom communication of sRNAs between fungi and plants

Plants and pathogens interact by secreting a variety of virulence factors known as microbe associated molecular patterns (MAMPs). These MAMPs are recognized by the MAMP receptors on plant cell surfaces, thereby inducing a defence response. However, many pathogens also secrete effector proteins into host cells that intercept defence signalling and induce pathogenesis [31]. These effectors are either apoplastic in nature and interact with the cell surface receptors or cytoplasmic in nature and are translocated inside the plant cell [32]. More than 80 effector proteins have been cloned and characterized from major crop-infecting fungi, the majority of which are encoded by avirulence (*Avr*) genes [33]. Recent research is beginning to reveal that fungal phytopathogens also secrete RNAs into their interacting host. This deployment of small RNA among two different interacting organisms, such as during the host–pathogen interaction, is termed as cross-kingdom sRNA interference (ckRNAi) [34]. ckRNAi was first reported when the black mould fungus *Botrytis cinerea* sRNA was detected in the target host plants *Arabidopsis thaliana* and *Solanum lycopersicum* [20]. Bc-sRNAs inhibited the AGO1 protein expression and silenced the plant immunity by creating a favourable condition for successful fungal colonization and the development of disease symptoms. These sRNAs therefore could act like a class of pathogen effectors that could regulate the host immunity by silencing the defence responsive genes in plants. In another study, Arabidopsis cells were found to secrete extracellular vesicles to transfer sRNAs into *Botrytis cinerea* that induced silencing of fungal genes critical to pathogenicity [10]. sRNA transfer from the pathogen to the host has also been reported in animal host–parasite interactions. For example, sRNA from the fungal pathogen *Beauveria bassiana* was transported into mosquitoes, resulting in the silencing of toll receptor spatzle protein 4 [35]. Asiatic rice stem borer, *Chilo suppressalis*, when fed with insect-specific miRNAs, miR-14 declined the expression of a host-specific spook (spo) and ecdysone receptor (EcR) gene, leading to large-scale mortality and developmental defects [36]. Thus, the mechanism of cross-kingdom RNAi mediated by pathogen derived sRNA not only plays a major role in the control of pathogenesis but also has a significant contribution in identifying disease-causing genes that could be used in the development of novel crop protection strategies [37].

### Plant sRNAs and their targets in fungi

Plants not only communicate endogenously through intracellular signalling pathways but also connect externally using various secretory molecules, hormones, volatile organic compounds, and sRNAs [38,39]. Plant-derived sRNAs play a major role in defence response mechanisms against various pathogenic organisms including fungi, water mould, and parasitic plants by inducing gene silencing mechanisms [40]. *Verticillium dahlia* is a soil-borne, hemibitrophic fungal pathogen responsible for devastating wilt diseases in many plants. sRNA sequencing of *V. dahlia* recovered from the infected cotton plants has revealed the presence of two cotton miRNAs, namely Ga-miR166 and Ga-miR159, in the pathogen [18] (Table 1). These miRNAs are responsible for inhibiting the virulence of *V. dahlia* by silencing the *Ca*^*2+*^ *-dependent cysteine protease calpain (clp-1)* and *isotrichodermin C-15 hydroxylase (HiC-15)* genes. C-15 hydroxylase, which produces trichothecene metabolites, and clp-1 are both involved in fungal phytopathogenicity [18]. *Arabidopsis thaliana* also exports sRNA to the necrotrophic fungus *Botrytis cinerea* and inhibits defence genes. Among the 17 Arabidopsis tetraspanin genes (TET1-TET17), TET-8 and TET-9 have shown increased expression following *B.* *cinerea* infection [41]. Cai et al. [10] reported that host sRNAs released into the apoplastic space are encapsulated in the TET-8 associated exosomes and transferred into *Botrytis cinerea* to inhibit the pathogenicity [10]. Arabidopsis also deploys endogenous transcript-derived siRNAs into *Phytophthora capsici* oomycetes to silence the virulence-related genes in the phytopathogen [42]. Inhibition of fungal pathogenesis has also been achieved through the spraying of double-stranded-noncoding small RNA (ds-sRNA) on the infected plant parts. For instance, small RNA-induced gene silencing (SIGS) has been performed by spraying a long noncoding dsRNA (791 nt *CYP3*-dsRNA) in barley infected with *Fusarium graminaream*. The dsRNA targets the fungal ergosterol gene, such as cytochrome P450 *lanosterol C-14α-demethylases*, causing inhibition of the fungal growth [28]. Overall, these evidences indicate that plant sRNAs are presumably translocated into fungal phytopathogens to inhibit the expression of virulence factors in the pathogen and augment the host defence response.

**Table 1.** Plant sRNAs as regulator of fungal pathogenicity.

Plant	Fungus	miRNA	Target gene	References
Cotton	*Verticillium dahliae*	miR166, miR159	clp-1, HiC-15	(Zhang et al., 2016)
*Arabidopsis thaliana*	*Botrytis cinerea*	TAS1c-siR483 TAS2-siR453	Bc-Vps51 Bc-DCTN1	(Cai et al., 2018)
*Arabidopsis thaliana*	*Phytophthora capsici*	At-SiRNAs	PSR2	(Hou et al., 2020)

### Fungal sRNAs and cross-kingdom RNAi

Recently, a wide range of pathogenic sRNAs have been reported to get translocated into host plants, causing loss of function of the host resistance through cross-species RNAi (Table 2). For instance, the necrotrophic fungal pathogen *B. cinerea* translocates sRNAs (Bc-sRNAs) to the target host plant and hijacks the defence and immune-responsive genes of the plant by suppressing RNAi machinery [20]. Bc-sRNA effectors detected in *Arabidopsis thaliana* and *Solanum lycopersicum* are Bc-siR3.1, Bc-siR3.2, Bc-siR5, and Bc-siR3.7, all of which are 21 nt in length and have a significantly higher count *in planta* as compared to cultured fungal biomass [20]. In *Arabidopsis*, Bc-siR3.2 targeted the genes encoding *mitogen-activated protein kinase-1(MPK-1)* and *mitogen-activated protein kinase-2 (MPK-2)*, Bc-siR3.1 targeted the oxidative stress-related gene encoding *peroxiredoxin (PRXIIF)*, and Bc-siR5 targeted the gene encoding a *cell wall-associated kinase (CAW)*. Similarly, Bc-siR37 targeted as many as 15 immunity-specific host genes encoding *WRKY7* transcription factor, protein kinases, FE12, PMR6 and RING-finger proteins [21]. Likewise, Bc-siR3.2 in *S. lycopersicum* also targeted the MPK signalling pathways, with the primary target being, *Sly-MPKKKK4* gene [21]. All these genes targeted by the fungal sRNAs are involved in defence signalling and host metabolism [20]. Thus, the translocated fungal sRNAs create a suitable environment for the growth of pathogens by silencing the host immune-responsive and growth-specific transcription factor genes. Notably, the pathogenic nature of the Bc-siR3.1, Bc-siR3.2, and Bc-siR5 depended on the function of the DCL protein of *B. cinerea* and they suppressed the cell’s RNAi mechanism by binding to the AGO protein of the host [21]. Interestingly, all the Bc-sRNAs originated from the long terminal repeat (LTR) transposon region [21]. LTRs are rapidly evolving groups of transposable elements and would be required for the co-evolution of RNAi mechanism [43] as required for host–pathogen interaction. In another study, *V. dahlia* sRNAs (*Vd-sRNAs*) were also reported to silence defence responsive genes in *Arabidopsis* by binding with the host AGO1 protein [44]. In a corroborative study, sRNA profiling detected a virulent specific precursor miRNA like RNA1 (VdMILR1) gene within the region of 2,083,943–2083870 of chromosome 8 of *V. dahliae* genome [22]. The matured *VdmiLR1* was synthesized through a DCL and AGO protein independent pathway and targeted a hypothetical virulence protein coding gene, *VdHy1* through increased H3K9 methylation at the 3’UTR region. Thus, the epigenetic regulation of the fungal virulence factor VdHy1 by *VdmiLR1* suggests a crucial role for sRNAs in the regulation of pathogenicity that can have a significant impact on pest management. In yet another recent study, sRNAs from the oomycete *Hyaloperonospora arabidopsidis* employ the host plant’s Argonaute (AGO)/RNA-induced silencing complex for virulence [24]. A genome-wide sRNA profiling revealed 133 unique sRNAs (Hpa-sRNAs) from *H. arabidopsidis*, 33 of which have their target sites in the Arabidopsis genome. Two *AtAGO1* enriched sRNA candidates, Hpa-sRNA2 and Hpa-sRNA90 were predicted to target the *WITH NO LYSINE (K) KINASE* (*AtWNK*)2 and *APOPLASTIC, ENHANCED DISEASE SUSCEPTIBILITY1-DEPENDENT (AtAED3)* genes, respectively. While *AtAED3* is implicated in systemic immunity, *AtWNK2* has been reported to be involved in flowering and the abiotic stress response [24]. This suggests that HpasRNAs exhibit cross-kingdom RNAi (ck-RNAi) by silencing plant genes that contribute to host immunity.

**Table 2.** Pathogenic sRNAs impairing host immune responses through cross-kingdom RNA interference.

Fungus	Plant	miRNA	Target gene	Reference
*Botrytis cinerea*	*Solanum lycopersicum*	Bc-siR3.2	MPKKKK4	Weiberg et al. 2013
*Botrytis cinerea*	*Arabidopsis thaliana*	Bc-siR3.2, Bc-siR3.1, Bc-siR5, Bc-siR37	MPK-1, MPK-2, PRXIIF, CAW, WRKY7, FE12, PMR6, RING	Wang et al. 2017
*Verticillium dahliae*	*Arabidopsis thaliana*	Vd-sRNAs	Defence responsive genes	Wang et al, 2016; Wang et al. 2017
*Verticellium dahliea*	*Gossypium herbaceum*	VdmilR1	VdHy1	Jin et al. 2019
*Fusarium graminearum*	*Triticum aestivum*	fg-sRNA1	TaCEBiP	Jian and Liang, 2019
*Hyaloperonospora arabidopsidis*	*Arabidopsis thaliana*	HpasRNA2, HpasRNA90	AtWNK2, AtAED3	Dunker et al. 2020
*Sclerotinia sclerotiorum*	*Arabidopsis thaliana*	Ssc-sRNA	SNAK2, SERK2	Derbyshire et al. 2019
*Phytopthera infestans*	*Solanum lycopersicum* *Solanum tuberosum*	Pi-sRNAs	StLL1	Hu et al. 2020
*Puccinia striiformis f sp. tritici*	*Triticum aestivum*	pst-milR1	PR2	Wang et al. 2017
*Blumeria graminis f. sp. horde*	*Triticum aestivum*	Bgh-sRNAs	Metabolic pathways	Kusch et al. 2018
*Blumeria graminis f. sp. tritici*	*Triticum aestivum*	Bgt-sRNAs	Metabolic pathways	Kusch et al. 2018
*Magnaporthe oryzae*	*Oryzae sativa*	milR236	MoHAT1	Li et al., 2020
*Penicillium italicum*	*Citrus cinensis*	milR7	AP2/B3	Yin. Et al.,2020
*Valsa mali*	*Malus domestica*	MilR16	*VmSNF1, VmDODA*, *VmHy1*	Xu et al., 2020
*Fusarium oxysporum f sp. lycopersici*	*Solanum lycopersicum*	Fol-milR1	FRG-4	Ji et al., 2021
*Pisolithus microcarpus*	*Eucalyptus grandis*	*Pmic_miR-8*	NB-ARC	Wong-Bajracharya et al., 2022

sRNA communication has also been reported from agriculturally significant phytopathogens infecting cereal crops. *Puccinia striiformis* f. sp. *tritici* (Pst), one of the most destructive pathogens of wheat. *In planta* sRNA profiling have detected a novel microRNA like RNA termed Pst-milRNA1 which targeted the wheat *pathogenesis-related 2 (PR2)* gene [21]. Pst-milR1 is 20 bp in length, starts with uridine, and specifically targets the poly-adenylated (Poly-A) region of the *beta-1,3 glucanase*, a *PR2* gene. Co-transformation and silencing of Pst-milR1 resulted in increased resistance, while the *PR2* knock-down plants demonstrated increased susceptibility to *Pst* in wheat. The biotrophic fungi *Blumeria graminis f. sp. hordei* (*Bgh*) and *Blumeria graminis f. sp. tritici* (*Bgt*) cause powdery mildew disease in barley and wheat, respectively, with an evolved RNAi machinery that expresses during infection [25]. A genome-wide sRNA profiling has revealed that 6 candidate sRNAs from *Bgt* and 15 from *Bgh* have solely predicted exclusive plant target genes and induced RNAi by potentially affecting various catabolic and plant transport systems during infection [25]. Likewise, the *Fusarium* head blight caused by *Fusarium graminearum* is another devastating disease of wheat that causes significant yield losses. sRNA profiling of the fungal phytopathogen has reported 264 pathogenic sRNAs that have predicted targets in the wheat genome [23]. Among others, Fg-sRNA1 specifically targeted a resistance related gene encoding *Chitin Elicitor Binding Protein (TaCEBiP)*. Western blotting and fluorescence observation have shown that Fg-sRNA1 suppressed the accumulation of *TaCEBiP in vitro* and enhanced the invasion of *F. graminearum* strain PH-1. This suggests that pathogenic sRNAs like Pst-milR1 and Fg-sRNA1 are important pathogenicity factor that act as effectors to suppress host immunity by silencing the resistance-related genes in wheat.

*Sclerotinia sclerotiorum* is an ascomycete fungus with a wide host range of more than 600 plant species. Derbyshire et al. [45] reported that *S. sclerotiorum* produces nearly 374 highly abundant sRNAs during infection of two of its prominent hosts, *Arabidopsis thaliana* and *Phaseolus vulgaris*. Most of these pathogenic sRNAs were predicted to target the host genes with functional domains associated with plant immunity and quantitative disease resistance (QDR). The silencing of two of the Arabidopsis target genes, *AtSERK2* and *AtSNAK2* resulted in enhanced susceptibility to *S. sclerotiorum* than the wild-type plants. While *At-SERK2* belongs to the brassinosteroid, PAMP, and DAMP signalling pathways, *AtSNAK2* is implicated in carbon signalling and systemic resistance [45]. This suggests that the cross-kingdom movement of *S. sclerotiorum* sRNA contributes to the silencing of immune components in host plants. In a similar fashion, *Phytophthora infestans* AGO1 associated sRNAs also exhibit cross-kingdom translocation during potato leaf infection [46]. Extensive target analysis of potato with pathogen derived sRNAs identified 648 sequences encoding for resistance (R) proteins. A single pathogen-specific miRNA (miR8788) was reported to target a potato *alpha/beta hydrolase-type encoding* gene (*StABH1*) encoding a membrane protein. The overexpression of *StABH1* in potato inhibited the infection, while the miR8788 knock out pathogenic strain showed growth of the pathogen in potato [46]. This suggests that the sRNAs encoded by *P. infestans* affect potato mRNA through cross-kingdom RNAi, further expanding our knowledge of the multifaceted strategies employed by this pathogen to facilitate infection.

sRNA communication has also been reported in fungal pathogens infecting fruits and vegetables. Citrus blue mould, caused by *Penicillium italicum (Pit)* is one of the most devastating pathogens of post-harvest citrus fruits, resulting in losses up of to 80%. The silencing of *Pit-DCL1* and *Pit-DCL2* has shown a variable response to disease development [47]. The *Pit-DCL2* RNAi transformants showed impaired pathogenicity as compared to *Pit-DCL1* RNAi lines or the wild-type strain. Interestingly, sRNA profiling detected 12 novel milRNAs, 4 of which have predicted targets encoding innate-immunity or biotic stress-related proteins. More precisely, Pit-novel 7 milRNA targeted the *AP2/B3* like transcription factor associated with plant immunity. This indicates that sRNA trafficking from *P. italicum* to citrus fruits was important in the molecular virulence mechanism during the P. *italicum*-citrus interaction. Likewise, *Valsa mali*, the necrotrophic ascomycete fungus that causes apple valsa canker disease have demonstrated adaptive regulation of virulence genes by milRNAs [48]. Vm-milR16 was reported as a significant contributor to pathogenicity by adaptively regulating the expression of *sucrose non-fermenting 1* (*VmSNF1*), *4,5-DOPA dioxygenase extradiol* (*VmDODA*), and a *hypothetical* (*VmHy1*) protein-coding gene. However, the cross-specific movement of Vm-milR16 or its expression *in planta* is yet to be established. Recent evidence also indicates that milRNAs from *Fusarium oxysporum* f. sp. *lycopersici* (Fol) interfere with tomato resistance during infection [26]. sRNA profiling revealed that Fol-milRNA1 is exported into tomato plants during infection, and its overexpression *in planta* led to enhanced virulence. Fol-milR1 targeted the tomato *calcineurin-B like* (*CBL*) protein kinase and inhibited its expression at the post-transcriptional level. Further, Fol-milR1 interfered with the host immunity machinery by binding to tomato AGO 4a. Taken together, all these studies clearly suggests that pathogenic sRNAs, are an important type of pathogenicity factor that contributes to impairing host immune responses through cross-kingdom RNA interference (ck-RNAi).

## sRNAs transport between plant and fungi through extracellular vesicles

**Figure 1.**
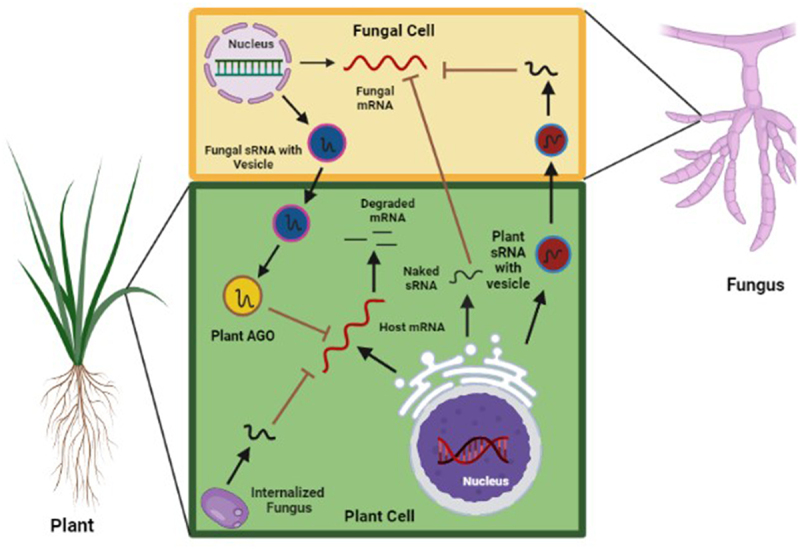
Cross kingdom RNA interference in plant–fungus interactions. Plant derived small RNAs (sRnas) can be packaged by Golgi and is efficiently absorbed by fungal cell which inhibits germination of spore and development of mycelia by cleaving the pathogenicity target gene. Fungal pathogen also deploy group of sRnas to plant to silences the host resistance gene. Plant use extracellular vesicle to transport small RNA into the fungal cell for inhibiting virulence related genes. The mechanism of transport for fungal sRnas is unclear.

Extracellular vesicles are membrane-bound vesicular compartments released by cells to the extracellular location to facilitate cell-to-cell transport of proteins, lipids and macromolecules, cellular invasion, and trafficking with the interacting organisms [49]. In animals, the EVs are classified as exosomes derived from the multivesicular bodies (MVBs) and the microvesicles derived from the plasma membrane that are involved in cell-to-cell communication [50]. EVs are uniquely positioned as vectors for cross-kingdom RNAi dissemination by protecting them from enzymatic degradation and providing opportunities for cell targeting [51]. Accumulated evidence and previously cited studies suggest that plant cells as well as fungi secrete different classes of EVs that have a significant role in sRNA trafficking [10]. In mammals, MVBs are enriched with tetraspanin (TET) proteins on their membrane, which are commonly used as biomarkers. During plant–fungus interaction, both plants and fungi traffic sRNA across each other for silencing the target genes through EVs [52]. When a plant deploys sRNAs to the fungus and inhibits their gene expression by targeting the pathogenesis and virulence-related genes, it is termed as HIGS, on the contrary when a fungus deploys small RNAs to the plant’s target genes and inhibits the gene expression is known as pathogen-induced gene silencing (PIGS) [53]. Interestingly, Arabidopsis has 17 members of the TET family proteins, two of which, *TET8* and *TET9*, are induced upon *B. cineria* infection [10]. *TET8* associated EVs in Arabidopsis are derived from MVBs and contain plant endogenous sRNAs that are taken up by *B. cinerea*. These exosomes deliver the plant-specific sRNAs into the fungal cell to suppress the infection by silencing the fungal pathogenicity-related genes [10]. During pathogenic infections, EVs are secreted from the Golgi apparatus and supplemented with proteins, lipids, coding RNAs, noncoding RNAs, and other constituents [54] (Figure 1). Likewise, the EVs are also used by *Arabidopsis* for the transport of secondary phasiRNAs into *Phytopthora capsici* to silence the target genes in the pathogen [40]. In a previous study, exosomes in the sunflower seedling were reported to transport sRNA to the fungal pathogen *Sclerotinia sclerotium*, leading to suppression of its virulence properties [17]. More recently, tomato exosomes significantly inhibited the germination of spores at the infection site after treatment with multiple fungal phytopathogens, including *B cinerea*, *Alternaria alternata*, and *Fusarium oxysporum* [16].

In addition to exosomes, the PENETRATION (PEN)1-associated EVs that contain several stress-related proteins have also been purified from the apoplast wash fluid of Arabidopsis leaves, suggesting that they could be associated with sRNA transport [55]. Baldrich et al. [56] reported the presence of tiny RNAs of 10 to 17 nt length in PEN-associated EVs, but their role in plant–pathogen interaction could not be ascertained. Arabidopsis PEN1 and PEN3 associated EVs were reported from the extracellular surrounding of the haustoria of the mildew fungus, *Golovinomyces orontii*, suggesting that they contribute to the defence response presumably through the transport of tiny RNAs [57]. Over and above these, a third type of plant EV derived from a double membrane-bound excocyst-positive organelle (EXPO) has been reported from the apoplastic region of the Arabidopsis cells [58]. However, their role in sRNA delivery or ck-RNAi is not clear. Nevertheless, the plants have adapted an EV-mediated ck-RNAi as part of an evolutionary defence mechanism for interacting with the fungal phytopathogens.

Secretion of EVs by fungi was reported in the 1960s. Hyphae of true fungi (Eumycota) secreted vesicles, termed lomasomes, that looked and behaved a lot like MVBs [59]. As in plants, the EV formation in fungi is also regulated by the endosomal sorting complex required for transport (ESCRT) [60]. These fungal MVBs were reported to fuse with the plasma membrane and release their content into the cell wall [61]. Analysis of *Aspergillus fumigatus* protoplasts has recently shown that specific EVs are generated through budding in the plasma membrane similar to the microvesicles [62]. An unconventional protein secretion pathway using EVs has also been reported in *Phytophthora infestans*, and in the rice blast fungus *Magnaporthe oryzae* to deliver effectors into the cytoplasm of their hosts [62]. As a large number of pathogenic sRNAs have been localized in plant cytoplasm, it is most likely possible that fungal EVs would be the carriers of the sRNAs to facilitate ck-RNAi in plants. However, additional studies are needed to fully interpret the mechanism of EV-mediated ck-RNAi during host–pathogen interaction.

## sRNAs transportation outside the extracellular vesicles

The experimental research discussed in the previous section suggests that the apoplastic sRNAs either get encapsulated in the EVs or tightly bound to the RNA binding proteins to avoid degradation [63]. However, the removal of EVs from the AWF does not always deplete the concentration of sRNAs, suggesting that EVs are not the primary location of these siRNAs and miRNAs [56]. This contradictory representation has been recently addressed through a seminal experiment by the Roger Innes group [11]. EVs collected from the AWF of Arabidopsis leaves, when treated with protease and RNAse A, eliminated the majority of sRNAs of size classes 21, 22, and 24 nt. Besides, the long non-coding RNAs (lnc-RNAs), including the circular RNAs (circRNAs) also form part of the extracellular RNAs enriched with the post-transcriptional modification of N6-methyadenine sites, meaning that they are stabilized by binding with glycin rich RNA binding proteins or the Argonaute protein [11]. While the lncRNAs have been implicated in multiple biological processes, including gene expression and stress response, their role in cell-to cell communication in the apoplast region is yet to be investigated. In animal system, the circRNAs have been reported from EV preparations recovered from cell culture media, suggesting that the excreted circRNAs could contribute to cellular communications [64]. Taken together, it is very alluring to speculate that the lncRNAs, including the circRNAs could be responsible for pathogenic sRNA sequestration in the apoplast before they are target delivered inside the host cell [11,65]. In other words, the extracellular RNAs located outside the EVs could also be responsible for HIGS. However, the exact nature of the non-EV sRNA transportation requires further investigation.

## Cross-kingdom sRNA communication for future agriculture

Phytopathogenic fungi are the major cause of yield losses in agriculturally important crops and affect global food security. Therefore, it is imperative to use all the alternative approaches, including the usage of sRNAs, to develop high-yielding resistant crop varieties for balanced nutrition and food security of an ever-increasing world population [66]. Both plants and animals require innovative and durable antimicrobial drugs, biodegradable fungicides, and insecticides to control pathogen infection. sRNA can transport between host and pathogen through cellular boundaries and therefore could be directly used for crop improvement. ck-RNAi-based direct application strategies, including HIGS, spray-induced gene silencing (SIGS), and virus-induced gene silencing (VIGS) have been recently recommended for crop protection [67].

### Host induced gene silencing

**Figure 2.**
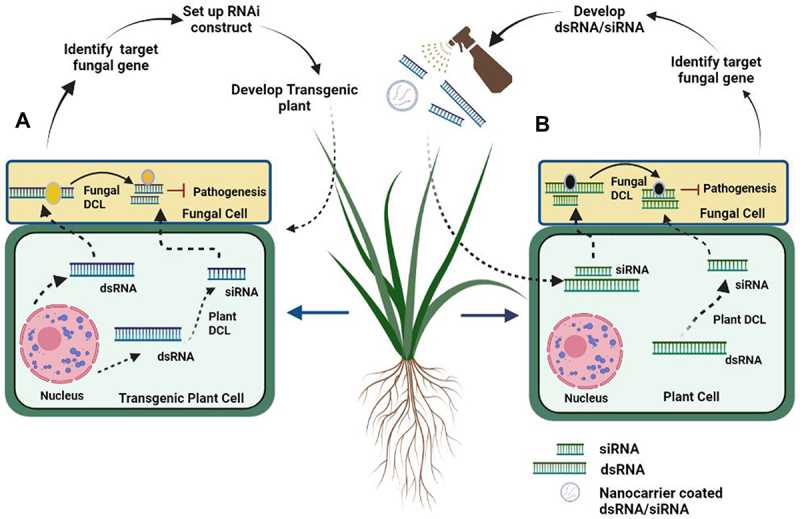
Strategies to prevent fungal infection by Host induced Gene Silencing (HIGS) and Spray Induced Gene Silencing (SIGS) A) HIGS pathway: Transgenic plant after producing dsRNA, undergo cleavage by using plant Dicer like (DCL), converting to small interfering RNA (siRNA). Both dsRNA and siRNA move to pathogen cell through plasma membrane silences virulence mRNA (target gene). B) SIGS Pathway: After construction of dsRNA/siRNA, are sprayed onto topical part of plant, which are directly taken by plant cell, by using plant Dicer like protein (DCL), dsRNA converted to siRNA. Both dsRNA and siRNA move towards fungal cell by plasma membrane and target fungal virulence mRNA.

HIGS is a promising RNAi-based technology in which genetically engineered plants express dsRNAs or sRNAs that target and silence virulence-related genes through RNAi to control plant diseases (Figure 2A) [68]. HIGS has proven as an efficient tool to combat various fungal phytopathogens in a wide range of plants through *in planta* expression of the RNAi construct. The fungal pathogens *Fusarium graminarium* and *Fusarium culmorum* are the major causative agents of *Fusarium* head blight in wheat and barley. *CYP3RNA*, a long dsRNA from transgenic Arabidopsis and barley, was used to target the genes involved in the ergosterol biosynthesis pathway, including *CYP51A, CYP51B*, and *CYP51C*, in the pathogen [69]. Suppression of fungal growth was reported after *in vitro* feeding of CYP3-dsRNA in both the host plants. Likewise, an sRNA mediated HIGS approach was used to target three virulence-related genes; *PtMAPK1* (MAP kinase), *PtCYC1* (cyclophilin), and *PtCNB1* (calcineurin B) from the wheat rust pathogen, *Puccinia triticina* [70]. Transgenic wheat lines with the hairpin RNAi demonstrated broad-spectrum resistance against not only *P. triticina* but also against two other forma specialis *Puccinia striiformis f. sp. tritici*, *Puccinia graminis f. sp. tritici*. HIGS-induced expression of a sRNA-based RNAi construct targeting the *Aspergillus flavus* alpha-amylase gene *Amy1* has resulted in decreased fungal colonization and aflatoxin accumulation in maize kernels [71]. In rice, *in vivo* HIGS techniques have been used to exhibit improved resistance to 11 strains of the rice blast fungus *Magnaporthe grisea* through the *in planta* expression of RNA hairpins targeting the pathogen-specific bZIP transcription factor *MoAP1* [72]. More recently, HIGS has been used to target two ergosterol biosynthetic genes, *ERG6* and *ERG11*, from *Fusarium oxysporum* (Foc), which causes Panama disease in bananas [73]. Transgenic bananas expressing the ERG6-RNAi and the ERG11-RNAi demonstrated enhanced resistance to the Foc TR4 strain. In yet another study, a bean pod mottle virus-based HIGS strategy was used to suppress the expression of at least three virulent-related genes from the Asian soybean rust (ASR) pathogen, *Phakopsora pachyrhizi*, leading to a significant reduction in ASR symptom development in soybean leaves [74]. All these studies suggest that sRNA-derived HIGS is an effective strategy of RNAi for reducing fungal infection in agriculturally important plants. Nevertheless, it needs to be practiced with caution. The success of HIGS depends on a greater quantity of siRNA transport between the two organisms. As such, HIGS is not an efficient approach against necrotrophic fungi as they absorb nutrients from a dead host, which could not supply sufficient siRNAs [65]. Also, HIGS-induced silencing of individual genes is inadequate to control the disease due to functional redundancy and partial deactivation of target proteins. Moreover, functional genomics has also shown that siRNAs often silence off-target genes [65]. The silencing of target genes in unintended organisms, saturation of RNAi machinery, and undesirable immune stimulation also contribute to the off-target effect of HIGS [75]. In addition, HIGS also faces technical difficulties with respect to certain tissues (fruits and roots) or poor siRNA uptake in case of soil-borne fungal pathogens [76]. Moreover, HIGS also involves high costs and requires regulatory clearances for the commercialization of transgenic lines. In fact, transgenic crops are currently not acceptable in major parts of the world, rendering them unusable for at least the next few years. In the near future, there will be a need for an upgrade of HIGS strategy with regard to target selection, the development of efficient transformation constructs, and stable transgenic systems to realize its full potential in crop protection.

### Spray Induced Gene Silencing (SIGS)

Similar to the environmental RNAi exhibited by some invertebrate systems, external long dsRNA as well as short sRNAs are taken up by fungal cells that induce silencing of fungal genes in a sequence specific manner [44]. This has resulted in the development of spray-induced gene silencing (SIGS), a non-transgenic RNAi approach [10,44]. This method involved the control of fungal growth and associated infection through the spray application of dsRNAs or sRNAs targeting fungal virulence-related genes (Figure 2B). Gray mould disease caused by the necrotrophic fungal pathogen *B. cinerea* affects the vegetables, fruits, flowers, and leaves of several plants. The SIGS-based application of dsRNA and sRNA suppresses the grey mould disease in all types of plant tissues by silencing the *B. cinerea DCL1* and *DCL2* genes [44]. In another recent study, exogenous applications of dsRNA in grape bunches significantly reduced the virulence of *B. cinerea* by silencing three prominent virulence-related genes-*BcCYP51, Bcchs1*, and *BcEF2* [77]. The direct high pressure spraying of dsRNA, sRNA, and siRNA on the leaves resulted in reduced lesion size after infection. SIGS also reduced post-harvest losses of vegetables (tomato, onion lettuce), fruits (grape, strawberry, apple), and flowers (rose) caused by B. cinerea infections, establishing a new class of synthetic fungicides [78]. Similarly, a preventive spray of CYP3-dsRNA on the leaf surface can regulate *Fusarium* head blight in barley and wheat by silencing the ergosterol biosynthetic genes [28]. What more, the foliar spray of dsRNA or siRNA has efficiently suppressed the infection by several fungal phytopathogens, including *B. cinerea*, *F. graminearum*, *S. sclerotiorum, Rhizoctonia solani, Zymoseptoria tritici*, *Aspergillus niger* and *Verticillium dahlia* [79]. All these reports suggest that SIGS mediated by dsRNA or sRNA is an effective and sustainable strategy for pre- and postharvest protection of plants against fungal infection. However, the commercial application of SIGS faces several hurdles, such as premature silencing of RNAi on the plant surface, large scale dsRNA production, and suitable RNA stabilizing agents for usage of SIGS in field conditions [80]. Therefore, the effectiveness of SIGS largely depends on the RNA uptake efficiency of the pathogen, and it is therefore important to address methods to overcome such a barrier.

### RNAi embedded nanoparticles

RNA, being highly unstable under extracellular conditions, is often encapsulated with artificial vesicles, lipidosomes, or nanocarriers to increase the efficiency of RNAi molecules [81]. Micro- or nano encapsulation provides a safe and stable environment for the RNAi method during the subsequent release of dsRNA or sRNA. Nanocarriers act as excellent vehicles for the transport of nucleic acid due to their extremely small size, which facilitates easy mobility across the cell wall and plasma membrane. An ideal nanocarrier should be biodegradable, require fewer organic compounds, biocompatible, non-toxic, and cost-effective. The exogenous spray of dsRNA and nanocarrier conjugates on the topical part of plants, could activate the RNAi in pathogens. The spray application of dsRNA conjugated with layered double hydroxide (LDH) nanoparticles increased the efficacy of antiviral protection in *Pisum sativum* and *Nicotiana benthamiana* [82]. In a similar study, Gurusamy and colleagues reported that dsRNA conjugated with chitosan nanoparticles (CNP) remained protected from degradation by endonucleases and resulted in significant knockdown of endogenous genes of the pathogen [83]. The CNP-dsRNA complexes were also found intact for up to 5 days on leaf surfaces and resulted in 100% mortality of infecting pests through silencing of *Helicoverpa armigera* virulence genes [84]. In a recent development, *Escherichia coli*-derived anucleated minicells were used for dsRNA encapsulation in SIGS [85]. The minicell-encapsulated dsRNA (ME-dsRNA) was shielded from RNAse degradation and remained stable on strawberry surfaces. Further, the ME-dsRNA selectively knocked down the pathogen-specific chitin synthase class III (*Chs3a*, *Chs3b*) and DICER-like proteins (*DCL1* and *DCL2*) genes of *Botryotinia fuckeliana* leading to inhibition of fungal growth in strawberry [85]. What’s more, a wide range of nanocarriers, including DNA nanostructure, carbon dots, gold nanoclusters, single-walled carbon nanotubes, and cell-penetrating peptide (CPP) have been developed in recent times that can be improvised for the encapsulation of RNAi molecules for specifically targeting fungal phytopathogens [81]. Hence, the accurate path for the uptake and release of externally applied dsRNA or siRNAs during plant-fungal interactions demands more study for practical application under field conditions.

## Conclusion and future prospects

The RNA-mediated cross-kingdom communication has been recently established in diverse plant systems, confirming that movable RNA is a key regulatory molecule governing host-fungal pathogen interactions. Fungal pathogens deliver sRNAs into plants to inhibit the host immunity, while the plant sRNAs are transported into the interacting pathogen to suppress their virulence. Evidence indicates that EVs are the carriers of sRNAs across the plant host to pathogens and vice versa. While a plethora of plant EVs have been employed in sRNA transport to fungi, the details mechanisms for fungal EV biogenesis, cellular secretion, loading of RNA into the EV, and biological functions are still unknown. Understanding the different classes of EVs and their ability for encapsulation of sRNA in fungi would be crucial for ck-RNAi. Contrary to this, the recent discovery that the majority of sRNAs in the apoplast reside outside the EVs has further contradicted the actual mechanism of sRNA translocation across the species. Further, the presence of RNA-binding proteins like AGO and GRP bound with the lncRNAs in the apoplast indicates their possible function in the secretion and/or stabilization of sRNAs. It will be worth investigating the role of these lncRNAs, especially the circRNAs, in cell-to-cell communication or immune responses through sRNA trafficking. Nevertheless, the current knowledge of ck-RNAi and fungal RNA has allowed for the development of disease control strategies like HIGS and SIGS in several agriculturally important pathosystems. However, HIGS and SIGS also suffer from technical difficulties in terms of RNA uptake, mechanisms of RNA translocation by pathogens, and off-target activities. The recent innovation in novel RNA delivery methods, including nanocarriers and artificial vesicles, has tremendously improved the stability, efficacy, and scalability of ck-RNAi under field conditions. It is possible that future agriculture will concentrate around the application of environmentally friendly RNA-based fungicides, and cross-kingdom communication of sRNA will form an important research direction for controlling eukaryotic pests and pathogens.

## Declaration

This research did not involve the use of any animal or human data or tissue
